# New Kids on the Block: Development and Assessment of a Multispecialty Fascia Iliaca Block Protocol and Training Program for Geriatric Hip Fracture in the Emergency Department

**DOI:** 10.7759/cureus.80560

**Published:** 2025-03-14

**Authors:** Jeffrey A Kramer, Caroline Shepherd, David Hess-Homeier, Jason Ochroch, Samir Mehta, Gwen Baraniecki-Zwil, Frances S Shofer, Nabil Elkassabany, Sheriza Hussain, Nova Panebianco

**Affiliations:** 1 Emergency Medicine, University of Pennsylvania, Philadelphia, USA; 2 Anesthesiology, University of Pennsylvania, Philadelphia, USA; 3 Orthopedics, University of Pennsylvania, Philadelphia, USA; 4 Anesthesiology, University of Virginia, Charlottesville, USA

**Keywords:** emergency medicine, fascia iliaca block, hip fracture, multispecialty nerve block program, nerve block, regional anesthesiology

## Abstract

Background

Hip fracture is a common presentation to emergency departments. Opioid-based medications are often used for analgesia but are associated with increased morbidity and mortality. Regional anesthesia for hip fractures can improve pain and other outcomes with minimal risk. The adoption of this procedure in the emergency department and perioperative space is low due to a lack of training and inadequate buy-in from consultants.

Methods

The Departments of Emergency Medicine, Anesthesiology, Orthopedic Surgery, Pharmacy, and Nursing collaborated to develop a multispecialty ultrasound-guided infrainguinal fascia iliaca block (FIB) protocol and training program at a large, urban, Level 1 trauma center. Training for emergency medicine physicians consisted of a one-half-hour lecture teaching the FIB technique, recognition and treatment of local anesthetic systemic toxicity (LAST), where to find the necessary equipment, and how to utilize the FIB order set and procedure note template in the electronic medical record. Learners then participated in a one-half-hour simulation session using a high-fidelity, inexpensive, do-it-yourself model. To assess the participants’ perceived knowledge and comfort with the FIB, we administered a survey to participants immediately before and after the training sessions.

Results

Prior to training, 4% (n = 48) of emergency medicine (EM) participants reported that they knew how to perform the block, and 2% felt comfortable doing so. After training, 100% of the participants reported knowing how to perform the block, and 92% felt confident performing the procedure. From March 2022 to June 2023, 37 FI blocks were performed in the emergency department (ED), representing 15% of the 249 hip fractures presenting to the ED during this time. Prior to the intervention, EM providers were not performing the block.

Conclusion

The utilization of nerve blocks in our geriatric hip fracture population increased dramatically and persistently with the institution of this protocol.

## Introduction

Each year, more than 300,000 older adults are hospitalized in the United States for geriatric hip fracture [1]. Rates vary between women and men, with an annual mean rate of hip fractures of 957.3/100,00 for women and 414.4/100,000 for men [2]. In-hospital mortality ranges from 1.5% to 3% [3-4]. The leading cause of death in these patients is respiratory failure, accounting for as much as 40% of in-hospital deaths [4,5]. Opioids are frequently used for analgesia, with as many as 95% of hip fracture patients receiving them during their admission [6]. Opioids cause respiratory depression and can lead to confusion, sedation, cognitive impairment, constipation, nausea, and vomiting. These effects are amplified in the elderly due to polypharmacy, a decrease in lean body mass, and reduced hepatic blood flow in older patients [7]. Pain is often undertreated in older patients, which worsens the risk of delirium and increases the length of hospital stay and time to ambulation [8,9]. Guidelines by the American Academy of Orthopedic Surgeons endorse "strong" recommendations to employ a multimodal analgesic regimen, including the use of a preoperative peripheral nerve block [10].

Regional anesthesia of the lower extremity is an effective adjunct or alternative to opioids and is recommended by several academic specialty societies [11,12]. Regional anesthesia has been shown to provide better analgesia with less opioid use and opioid-related adverse effects, improved short- and long-term ambulation scores, and higher patient satisfaction [13-15]. The feasibility of emergency medicine (EM) physicians performing nerve blocks for hip fracture in the emergency department (ED) has been documented and shown to reduce the need for parenteral and oral opioids [16,17].

Despite this, nerve blocks have been significantly underutilized. A recent retrospective cohort study found that only 3.0% of hip fracture patients received a nerve block between 2004 and 2016 [18]. Common barriers identified in the EM and anesthesia literature were protocol familiarity, lack of training and procedural comfort, busy schedules, and staffing bandwidth [19-21]. Coordinated care pathways using standardized order sets, co-management protocols, and structured clinical pathways have shown improved outcomes in patients with hip fracture [22]. Similarly, multiple specialties are often involved, and standardizing care across specialties can improve efficiency, safety, and patient-centered communications.

The goal of this study was to describe a collaborative initiative to develop and evaluate a regional nerve block program for acute geriatric hip fracture at a single, academic, urban medical center, and demonstrate the feasibility of uptraining EM and anesthesia physicians on the infracinguinal fascia iliaca block (FIB) and building a long-lasting culture of nerve block utilization. We evaluated the initiative’s impact on EM physicians’ perceived knowledge and comfort with the FIB to determine if such a training program would be feasible. Despite prior publications describing the utility of the FIB for acute hip fracture in the ED, this is the first study, to our knowledge, that describes the process of multispecialty collaboration in the development of an ED FIB program and surveys physicians directly about their perceptions of the initiative.

## Materials and methods

Initiation and protocol development

The Departments of Emergency Medicine, Anesthesiology, and Orthopedic Surgery collaborated to develop a multispecialty ultrasound-guided FIB program at a large, urban, level 1 trauma center that sees over 47,000 ED visits a year. Approximately 175 patients with geriatric hip fractures are treated annually at this institution. Prior to the initiation of this program, no patients were receiving nerve blocks for hip fracture in the ED, and only 10.4% of patients were receiving a block by anesthesia in the perioperative space.

To start, the EM Ultrasound Site Director reached out to the Division of Regional Anesthesia and the Departments of Orthopedic Surgery, Pharmacy, and Nursing to identify partners in this initiative. We systematically targeted the pre-identified barriers to implementation, a lack of available medications, EM provider training, and multidisciplinary meetings with key stakeholders. A shared vision to improve patient care was required to eliminate these barriers. A regional anesthesia task force was established with the aim to connect key partners and provide a platform to communicate regularly. 

The FIB was chosen given its effectiveness in hip fracture pain control, relatively few contraindications, low complexity, and low risk of complications, including local anesthetic systemic toxicity (LAST) [17,23]. In order to maintain uniformity across departments, the FIB technique adopted by the ED mirrored that performed by anesthesia at the study site. Utilizing an infrainguinal approach, the FIB was performed under ultrasound guidance in an in-plane fashion. Thirty milliliters (ml) of ropivacaine 0.2% with 4 milligrams (mg) of preservative-free dexamethasone were injected using a nerve block needle (B. Braun 4” 20 GA Stimuplex® Ultra 360®). Prior to the block initiative, the ED did not have access to Ropivacaine or stock intralipid. These deficiencies were addressed as part of the initiative with pharmacy and anesthesia.

For the block, patients were placed on cardiac and oxygen saturation monitoring during and 30 minutes after the procedure. The ED nursing staff received in-service training on the recognition of signs and symptoms of LAST. An order set was created in the electronic medical record (EMR, EPIC, Verona, WI), which contained all medications utilized in the block and nursing instructions. Using a skin marker, the blocked leg was labeled with medication, dose, date, and time of block to ensure the following providers are aware of the treatment received. A block box was created, which contained nerve block needles, sterile ultrasound probe covers and gel, skin markers, and syringes. This could be brought to the bedside and minimize time searching for supplies.

Once a patient was diagnosed with a hip fracture, the orthopedic surgery team was notified that the ED staff would be performing a block on the patient. The orthopedic resident then performed a brief, expedited neurovascular exam. If the orthopedics resident was not available, the ED staff performed this task and documented the findings. A FIB procedure note template was developed in the EMR for rapid documentation of the procedure. To improve access to the FIB protocol and LAST treatment guidelines, laminated cards with this documentation were attached to the ultrasound machines, and a tutorial slide deck and links to training videos were placed on the ED’s intranet website.

Training program

A training program was created to teach the FIB to ED attendings, clinical ultrasound fellows, and EM residents. This consisted of a one-half-hour lecture teaching the FIB technique, the recognition and treatment of LAST, where to find the necessary equipment, and how to utilize the FIB order set and procedure note template in the EMR. Following the lecture, learners participated in a one-half-hour simulation session using a high-fidelity, inexpensive, do-it-yourself model.

**Figure 1:**
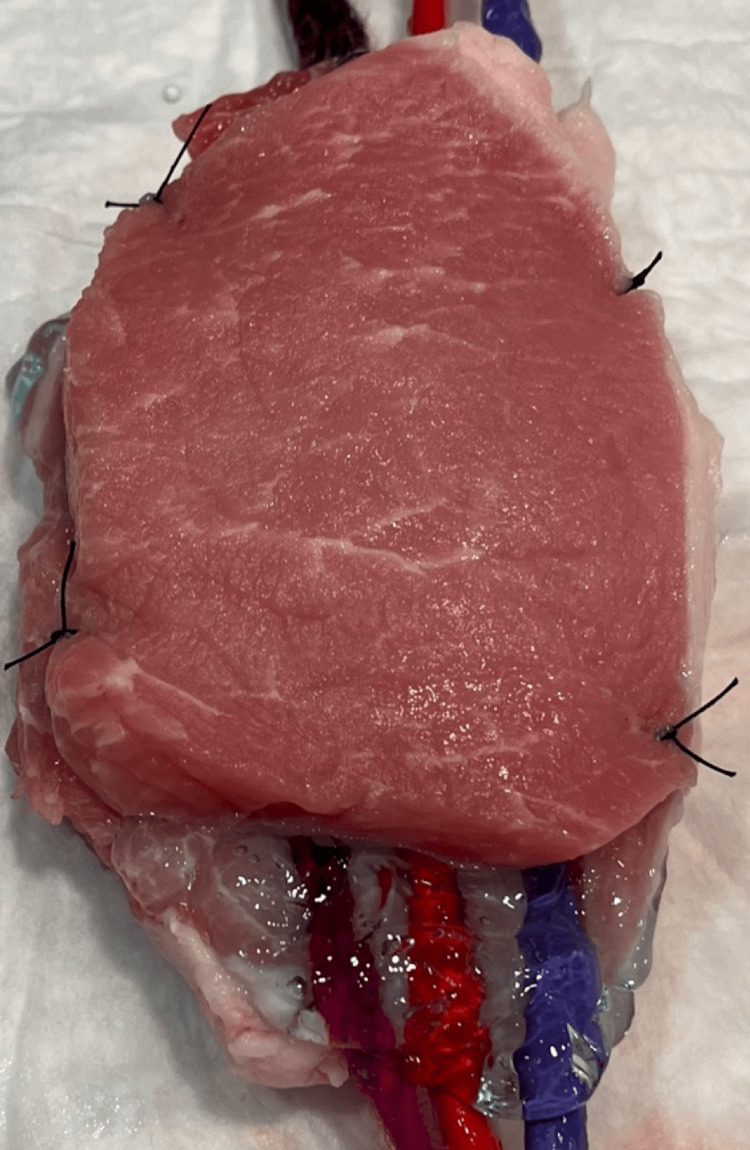
Simulation model created from two pork chops with gel-soaked yarn for nerve and water-filled animal balloons to simulate vessels.

We created a FIB simulation model out of two pork chops using a thin balloon filled with water for a vessel with an adjacent nerve made from yarn soaked in ultrasound gel. Grooves were cut into the pork chop in which to lay the balloon and yarn. The second pork chop was then placed on top of this, and the two pieces were sewn together to prevent them from sliding apart and introducing air artifact. This created a fascia layer with the simulated nerve and vessel situated in the fascial plane (Figure 1).

**Figure 2:**
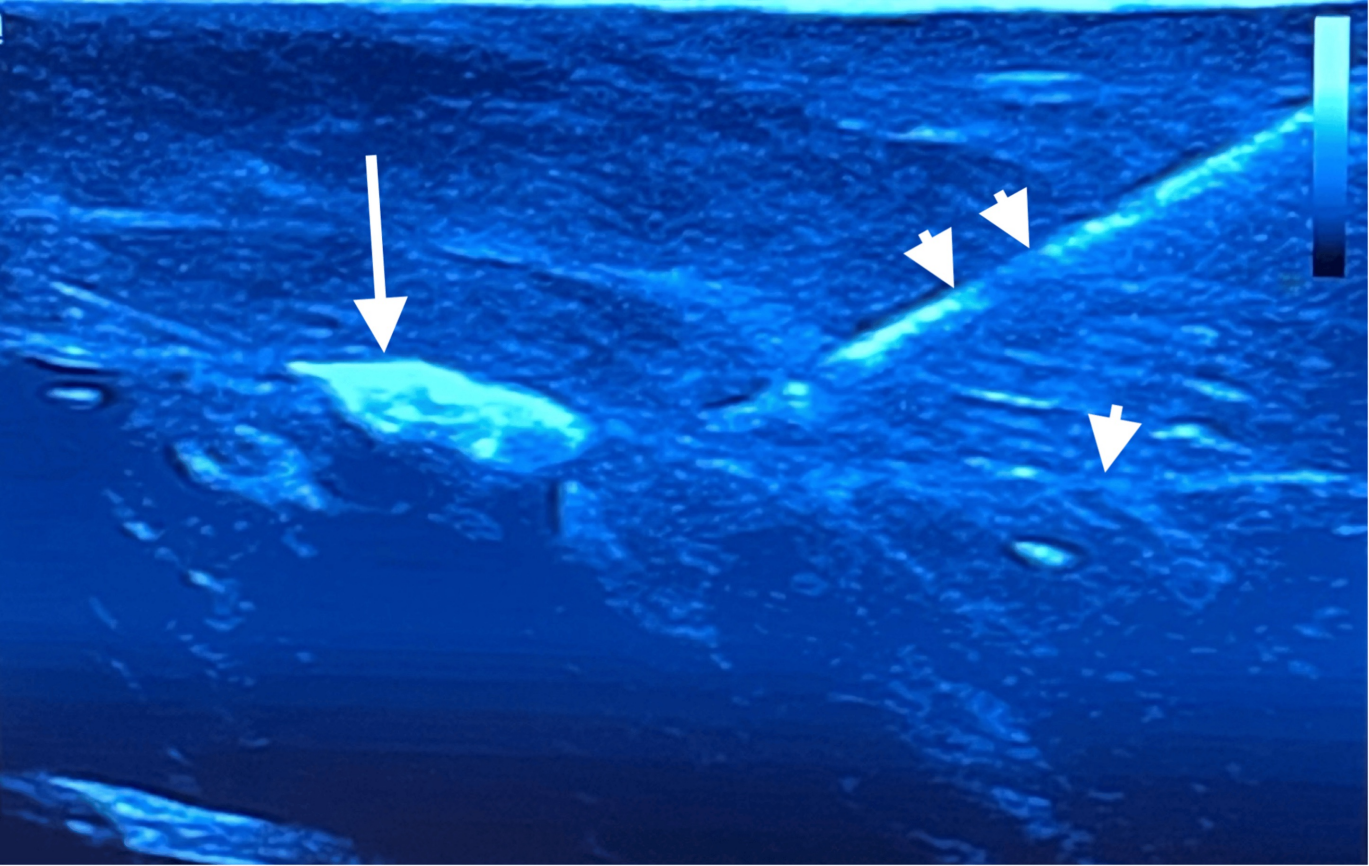
Still ultrasound image of simulation model displaying nerve (arrow), fascial plane (arrow head), and nerve block needle (double-arrow heads).

We found that natural fiber yarn created a much more realistic nerve than synthetic fiber yarn. Using nerve block needles, participants were taught how to follow the needle into the simulated facial plane under ultrasound guidance and hydrodissect the tissue layers (Figure 2). The participants were encouraged to practice the technique on the model until they felt comfortable with it. 

The anesthesia department provided educational support at the training sessions and invited EM attendings and clinical ultrasound fellows to shadow them while on service.

To assess the participants’ perceived knowledge and comfort with the FIB, we administered a survey to participants immediately before and after the training sessions. The survey included 12 questions in three areas: procedure (six), workflow (four), and recognition and treatment of LAST (two). The six questions assessing knowledge of the FIB procedure included; 1) whether participants knew how to perform the block, 2) if they were comfortable performing the block, 3) assessing the femoral nerve and surrounding anatomy using ultrasound, 4) assessing the fascia iliaca, and were they familiar with 5) the indications and 6) contraindications for the FIB. Four questions pertaining to the procedure’s workflow asked if the participants were familiar with the medications used to perform the FIB, if they knew what equipment was needed for performing the FIB, where they could find this equipment, and if they knew how to document the FIB. The participants were also asked if they were able to recognize the signs and symptoms of LAST and if they knew how to treat LAST (Table 1).

**Table 1:** Categories with their respective questions ^1^ fascia iliaca block, ^2^ local anesthetic systemic toxicity

Category	Question
Procedure	Know how to perform a FIB^1^
	Comfortable performing the FIB
	Comfortable assessing femoral nerve and surrounding anatomy using ultrasound
	Comfortable identifying the fascia iliaca
	Familiar with indications for FIB
	Familiar with contraindications for FIB
Workflow	Familiar with medications used to perform FIB
	Know what equipment needed for performing a FIB
	Know where to find equipment needed for a FIB
	Know how to document the FIB
Recognition and Rx of LAST^2^	Familiar with signs and symptoms of LAST
	Know how to treat LAST

For each question, the participants’ responses were assessed using a four-point Likert scale, with 1 = strongly disagree to 4 = strongly agree. The survey was conducted using Qualtrics (Provo, UT), which participants accessed via a quick response (QR) code on their cell phones. For each of the 13 questions, Wilcoxon sign-rank tests were used to assess differences pre/post-training. In addition, three scores were created (knowledge and comfort with FIB, understanding of workflow, and recognition and treatment of LAST) (Table 2) by dividing the points received pre and post by the total number possible, with higher scores indicating greater knowledge, comfort, and familiarity with FIB. The study was given exempt status through the University of Pennsylvania’s Institutional Review Board. Participation in the brief questionnaire was voluntary.

**Table 2:** Scores pertaining to knowledge and comfort with the FIB, understanding of workflow, and recognition and treatment of LAST ^1^ Scores converted to percentages: total points/total possible points. Higher percentages indicate greater agreement with statements. ^2^ All differences statistically significant at p < 0.0001. ^3 ^Local anesthetic systemic toxicity. FIB: fascia iliaca block, LAST: local anesthetic systemic toxicity

Category	Score range^1^	Pre-training	Post-training	D^2^
Procedure	6-24	47.5	86.9	40.2
Workflow	4-16	48.8	88.3	39.3
Recognition and Rx of LAST^3^	2-8	53.7	87.5	37.1

Statistical analysis

To determine differences in these scores pre/post, a paired t-test was used. To determine differences in these scores by participant training level, a two-factor analysis of variance in repeated measures was performed where training level (attending/fellow vs resident) was a fixed effect and time (pre/post survey) was the repeated measure. All analyses were performed using SAS statistical software (version 9.4, SAS Institute, Cary, NC). Figures were created using GraphPad Prism (version 9.5.1, GraphPad Software, San Diego, CA, USA). 

Anesthesia

As part of our multidisciplinary effort, the Department of Anesthesiology simultaneously implemented a protocol to target pre-identified barriers in the perioperative space. This included buy-in from the Orthopedic and Geriatric Departments to block all hip fracture types and surgical repairs and establishing expectations with periodic emails and resident orientations. The orthopedic hip fracture admission order set was customized to include a consult to the Regional Anesthesia and Acute Pain Service for the impending surgery and a block request. Similar to the EM protocol, the anesthesia providers also had laminated protocols on their US machines and easy access to video tutorials and pre-made supply kits.

## Results

A total of 24 EM attendings, 22 EM residents, and five clinical ultrasound fellows participated in the training. Of these, 48 completed both pre- and post-surveys. Pre-training regional anesthesia experience was low, with only three out of 48 (6%) participants reporting having performed the FIB, and only one (2%) participant reported comfort with the procedure, whereas post-training, 44 participants (92%) reported comfort with the block.

Our assessment of the three main categories of our program, knowledge of the FIB, understanding of workflow surrounding the procedure, and the recognition and treatment of LAST (Table 1), showed an increase in scores in each section. Knowledge of the FIB went from a score of 47.5 pre-training to 86.9 post-training (difference: 40.2, 95% CI: 35.2-45.2, p < 0.0001). Familiarity with the workflow for the FIB increased from 48.8 pre to 88.3 post-training (difference: 39.3, 95% CI: 33.5-45.2, p < 0.0001). Similarly, recognition and treatment scores of LAST increased from 53.7 pre to 87.5 post-session (difference: 35.4, 95% CI: 29.0-41.8, p < 0.0001).

**Figure 3:**
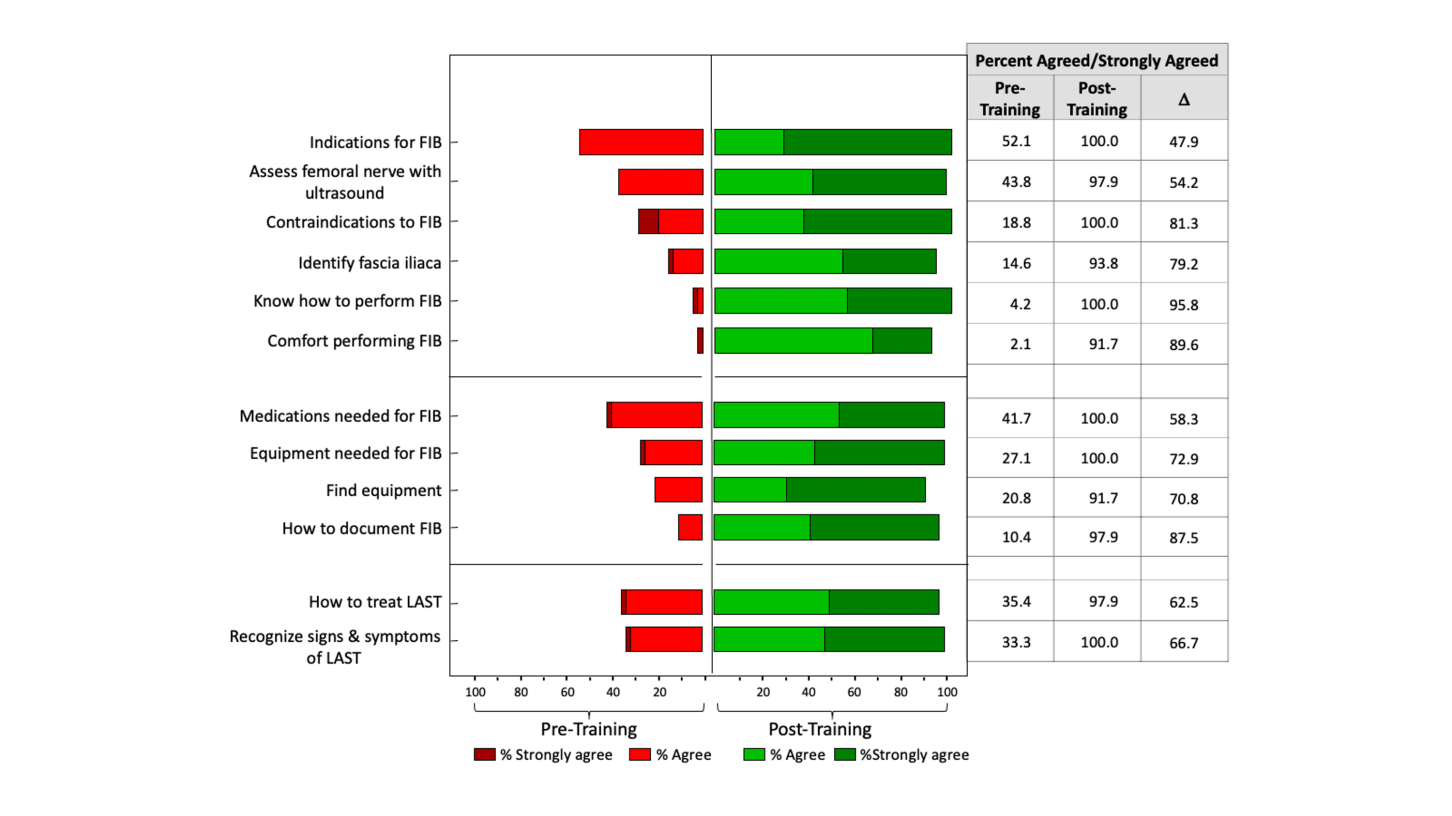
Pre- and post-training responses

Similar patterns were also seen for the individual questions. Post-training, 91-100% of the time, the participants agreed or strongly agreed with each of the 12 statements, whereas pre-training, the percentage who agreed/strongly agreed ranged from 2% to 52% (Figure 3).

**Figure 4:**
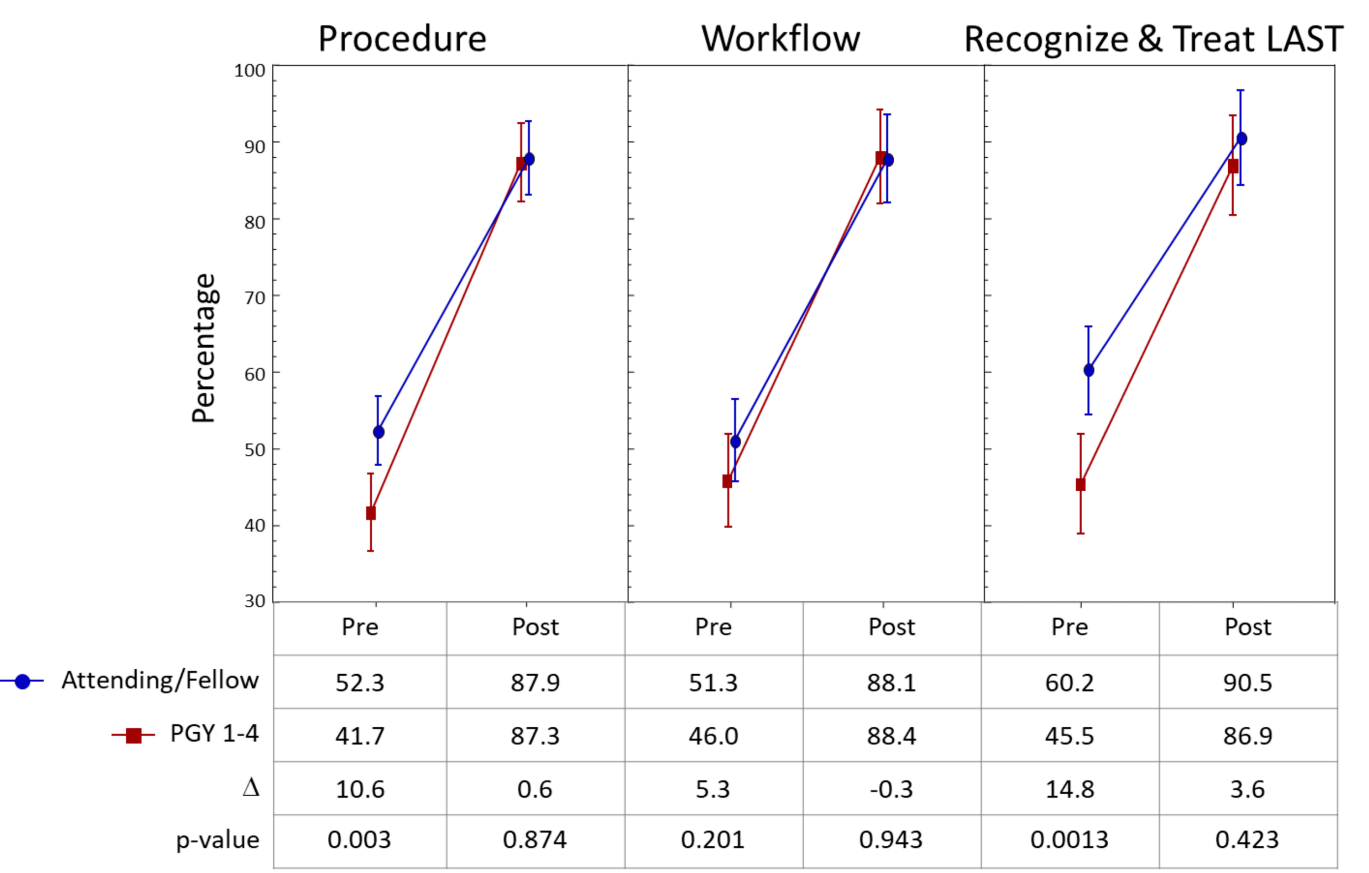
Pre- and post-assessment by level of training

There were significant differences in knowledge of the FIB procedure and recognition and treatment of LAST between residents and attendings/fellows, with the latter expressing more knowledge with the block and familiarity with LAST prior to training. After the session, this difference was no longer present on the post-training assessment, with both groups having increased scores on these items to approximately the same level (Figure 4).

All but one participant (2%) felt that the simulation model was an adequate simulation tool. After training, all participants agreed or strongly agreed that the educational materials adequately taught them how to perform the FIB procedure, that they could identify and treat LAST, that they were confident to perform the procedure, and that they had awareness of the equipment needed.

Objectively, between the EM and anesthesia initiatives, nerve block utilization rates improved dramatically. Baseline rates at our institution were at 10.4% pre-intervention, and for the first 18 months of initial interventions, they have risen to 38.4%. From March 2022 to June 2023, 37 FI blocks were performed in the ED, representing 15% of the 249 hip fractures presenting to the ED during this time. Prior to the intervention, EM providers were not performing the block. There were no complications reported with the blocks performed by the EM providers. 

## Discussion

In this manuscript, we describe the process of developing a multi-specialty-approved regional anesthesia pathway and report on trainees’ perception of the training program, the homemade, low-cost, nerve block simulator, and the program’s effect on confidence and knowledge of the procedure. We found a significant improvement in knowledge, understanding of workflow, and recognition and treatment of LAST after a multidisciplinary training initiative. Baseline rates of FIB at our institution increased over three-fold for the first 18 months of initial interventions. We expect that with continued up-training in the ED and building into the culture, these rates will continue to rise.

Opioids are commonly used in patients with geriatric hip fracture [6]. However, they cause respiratory depression, which is the leading cause of death in this population [4,5]. In addition, opioids can cause confusion, sedation, constipation, and cognitive impairment, which influence morbidity and mortality [7]. Regional anesthesia of the lower extremity is an essential component of multimodal analgesia in geriatric hip fracture patients; however, adoption has lagged behind traditional means of care [18,24-26]. 

Prior studies have described ED-based regional anesthesia initiatives for acute hip fracture but have neglected the need for multi-specialty investment and buy-in [25,27]. Our training program was unique in that we were able to collaborate with orthopedic surgery to secure urgent evaluation before the FIB and aligned with the department of anesthesia to augment training opportunities. In addition, we leveraged our highly engaged pharmacy colleagues to ensure appropriate medications were available in the ED and nursing to recognize the signs of LAST and monitoring necessary for patients receiving the FIB. 

Our training program focused on educating participants on how to perform the FIB, the workflow associated with the block, and how to recognize and treat LAST. Pre- and post-evaluation of these areas of knowledge showed a significant increase in scores. Attendings and fellows reported higher levels of knowledge than residents prior to the training. This difference was no longer present after the program.

Simulation is a valuable tool when teaching skills that are high-risk or rare [28]. The fidelity of a simulator is defined by how well it recreates the condition being mimicked. High-fidelity simulators are often expensive, bulky, and difficult to access because of competition for the resource. In our study, all but one participant felt that the pork model adequately mimicked the nerve/vessel/tissue of a human lower extremity and each simulator cost less than $20 USD to produce. In the scenario where scanning on raw meat is undesirable, unobtainable, or limited because of a lack of refrigeration, construction materials, or socio-cultural belief systems, other simulation models have been described [29].

Despite the evidence that regional anesthesia reduces the need for opioid analgesics, decreases time to mobilization, and reduces serious complications such as respiratory failure, it is not commonplace in emergency care. This study demonstrates that the development of a multi-specialty FIB program and EM FIB training is feasible and had a significant positive impact on EM providers’ knowledge of and comfort with the FIB and improved understanding of recognition and treatment of LAST.

Limitations

This study was limited in that it was conducted in the United States at a single, urban, academic center with more than 20 years of continuous fellowship and residency ultrasound education. By the nature of the EM training, most participants had extensive experience with ultrasound-guided procedures. It is possible that, in places with less robust ultrasound experience, the participants’ confidence post-training wouldn’t be as profound. Similarly, the non-EM specialties at our institution had awareness of the long-established ED ultrasound training program and has a point-of-care (POCUS) alliance committee with over 50 department ultrasound champions that meets monthly, which may have made multi-specialty collaboration more natural. The assessment tool utilized has not been validated, however, there is a lack of validated assessment tools for skills related to the performance of US-guided regional blocks. The assessment was mainly subjective and based on learner perception and not on the skills acquired during training. There is a possibility that the increase in scores on the tests may be partly due to the learning effect of the assessment tool, particularly given the short period of time between the administration of the test before and after the training session. 

## Conclusions

In this manuscript, we describe an elegant multispecialty collaboration between emergency medicine, orthopedic surgery, anesthesia, pharmacy, and nursing to develop a FIB protocol and training pathway for the analgesic management of geriatric patients with hip fracture. After a brief training session, the participants reported a significant increase in knowledge of how to perform the block and comfort with this skill. They felt the educational materials and simulation model adequately trained them for this endeavor. In addition, the participants felt that their ability to recognize and treat LAST improved, and all reported that they felt confident to perform the block after this training.
